# Neuroblastoma in dialog with its stroma: NTRK1 is a regulator of cellular cross-talk with Schwann cells

**DOI:** 10.18632/oncotarget.2611

**Published:** 2014-10-21

**Authors:** Kristian W. Pajtler, Ellen Mahlow, Andrea Odersky, Sven Lindner, Harald Stephan, Ivo Bendix, Angelika Eggert, Alexander Schramm, Johannes H. Schulte

**Affiliations:** ^1^ Department of Pediatric Oncology and Hematology, University Children's Hospital Essen, Essen, Germany; ^2^ German Cancer Research Center (DKFZ), Heidelberg, Germany; ^3^ Department of Peditrics I/Neonatology, University Children`s Hospital Essen, Essen, Germany; ^4^ Department of Pediatric Oncology/Hematology, Charité-Universitätsmedizin Berlin, Germany; ^5^ German Cancer Consortium (DKTK), Germany; ^6^ Translational Neuro-Oncology, West German Cancer Center, University Hospital Essen, University Duisburg-Essen, Essen, Germany; ^7^ Centre for Medical Biotechnology, University Duisburg-Essen, Essen, Germany

**Keywords:** Neuroblastoma, NTRK1, Schwann cells, migration, differentiation, NRG1

## Abstract

In neuroblastoma, the most common solid tumor of childhood, excellent prognosis is associated with extensive Schwann cell (SC) content and high-level expression of the neurotrophin receptor, NTRK1/TrkA, which is known to mediate neuroblastoma cell differentiation. We hypothesized that both stromal composition and neuroblastic differentiation are based on bidirectional neuroblastoma-SC interaction. Reanalysis of microarray data from human SY5Y neuroblastoma cells stably transfected with either NTRK1 or NTRK2 revealed upregulation of the mRNA for the SC growth factor, NRG1, in NTRK1-positive cells. Media conditioned by NTRK1-expressing neuroblastoma cells induced SC proliferation and migration, while antibody-based NRG1 neutralization significantly decreased these effects. Vice versa, NRG1-stimulated SC secreted the NTRK1-specific ligand, NGF. SC-conditioned medium activated the NTRK1 receptor in a neuroblastoma cell culture model conditionally expressing NTRK1 and induced differentiation markers in NTRK1-expressing cells. NTRK1 induction in neuroblastoma xenografts mixed with primary SC also significantly reduced tumor growth in vivo. We propose a model for NTRK1-mediated and NRG1-dependent attraction of adjacent SC, which in turn induce neuroblastic differentiation by secretion of the NTRK1-specific ligand, NGF. These findings have implications for understanding the mature and less malignant neuroblastoma phenotype associated with NTRK1 expression, and could assist the development of new therapeutic strategies for neuroblastoma differentiation.

## INTRODUCTION

Peripheral neuroblastic tumors are a family of common solid tumors of childhood. They are designated neuroblastomas, ganglioneuroblastomas or ganglioneuromas, depending on the level of neuroblastic differentiation, content of stromal cells and other specific features [1].

These tumors originate from primitive cells of the sympathetic nervous system, and their morphological features appear to recapitulate developmental stages of sympathetic ganglionic cells. Neuroblastomas may regress spontaneously, particularly in infants, or differentiate into ganglioneuroblastomas or benign ganglioneuromas. The prognosis of some children diagnosed with neuroblastoma over 1 year of age can be good, however, most of these older patients have developed metastases at diagnosis and have poor prognoses [2]. Neuroblastic tumors mainly consist of neuroblastic cells at different levels of differentiation and harboring variable quantities of Schwann cells in their stroma. Assessment of histopathological characteristics of these tumors allows assignment of patients to subgroups with favorable or unfavorable prognosis. Prognosis is poor for patients with undifferentiated neuroblastoma subtypes, with a 5-year overall survival of only 50%, while 5-year overall survival improves slightly for patients with poorly differentiated tumors to 69%, representing an intermediate prognosis. The preponderance of neuroblastic differentiation in the differentiating subtype is an indicator of favorable prognosis, and the 5-year overall survival increases to 87.3% in this group [3]. Interestingly, the quantity of Schwannian stroma directly correlates with tumor maturation and low vascularity [4, 5]. Thus, prognostication based on intratumoral Schwann cell content reveals excellent outcome for patients with Schwann cell stroma-rich (intermixed ganglioneuroblastoma) and stroma-dominant (well-differentiated ganglioneuroblastoma) tumors. Nodular ganglioneuroblastomas (composite tumors), which only contain islands of neuroblastic differentiation with schwannian stroma-poor areas, have intermediate prognoses [6].

Neuroblastic tumors are characterized by a broad biological heterogeneity, which can include amplifications of the *MYCN* and *ALK* oncogenes, allelic losses of chromosomes 1p, 3p and 11q, alterations of ploidy and dysregulated expression of neurotrophin receptors, each of which influencing clinical outcome to varying degrees [7]. Tyrosine kinase receptor signaling is a contributing biological factor to the diverse clinical spectrum observed in neuroblastoma patients. Activation of the neurotrophic tyrosine kinase type 1 receptor, NTRK1, by binding of the specific ligand, nerve growth factor (NGF), inhibits angiogenesis, induces differentiation and growth arrest and mediates apoptosis [8, 9]. In contrast, high intratumoral expression of NTRK2 and its specific ligand, brain-derived neurotrophic factor (BDNF), enhances proliferation, metastatic behavior and chemoresistance in neuroblastoma cells [10]. Remarkably, NTRK1 expression is highly correlated with the morphology of neuroblastic tumors, since tumors with favorable histologies express significantly higher levels of NTRK1 than those with unfavorable histologies [11].

In recent years, numerous studies have emphasized the importance of cross-talk between malignant tumor cells with their associated microenvironment, consisting of extracellular matrix, immune cells, tumor-associated vasculature and adjacent stroma [12, 13]. Stromal cells were demonstrated to promote neoplastic transformation of epithelial cells, to enhance tumor growth and to stimulate angiogenesis and metastasis by interaction with other stromal components [14, 15]. Evidence is mounting that tumor-stroma interactions in neuroblastomas might also contribute to a less malignant phenotype caused by increased tumor cell differentiation, reduced angiogenesis and a more efficient immunological tumor surveillance [16, 17]. The underlying molecular mechanisms and potential paracrine signals are, however, poorly understood.

Based on observations that i) Schwannian stromal cells are the predominant morphological features of favorable tumors and ii) NTRK1 expression is one of their major molecular characteristics, we hypothesized that both Schwannian stroma development and neuroblastic differentiation rely on bidirectional interactions. Here we analyzed expression patterns of Schwann cell stimulating factors in both cultured neuroblastoma cells and primary tumors. We further investigated the biological mechanisms underlying the postulated interactions between neuroblastoma and stromal cells using neuroblastoma cell lines with stable or inducible NTRK1 expression and primary Schwann cell cultures. Finally, we assessed the effects of NTRK1 expression in neuroblastoma cells on neuroblastic tumor progression in the presence of Schwann cells *in vivo*.

## RESULTS

### NTRK1 causes upregulation and secretion of NRG1

**Figure 1:**
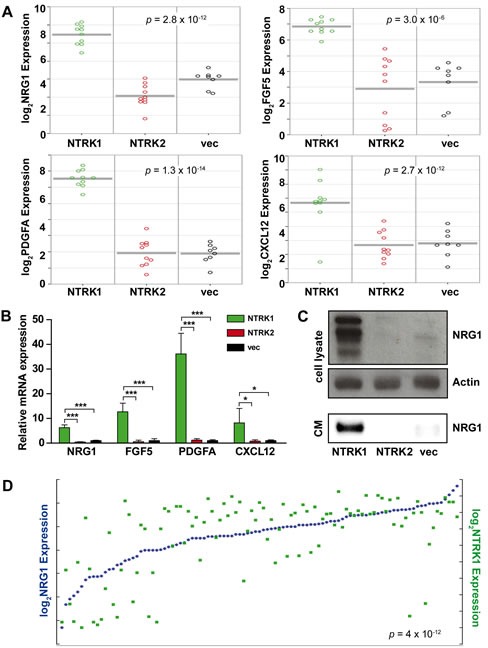
NTRK1 expression in neuroblastoma cells causes NRG1 upregulation and secretion *in vitro* and is strongly positively correlated with NRG1 expression *in vivo* (A) Graphs display *NRG1*, *FGF5*, *PDGFA* and *CXCL12* expression in SY5Y-NTRK1 (green circles), SY5Y-NTRK2 (red circles) and SY5Y-vec (black circles) cells. The R2 platform was used to extract data from previously obtained microarray analyses [18]. (B) Bars represent expression of *NRG1*, *FGF5*, *PDGFA* and *CXCL12* measured using real-time RT-PCR and normalized to the geometric mean of GAPDH, UBC and HPRT expression in SY5Y-NTRK1 (green), SY5Y-NTRK2 (red) and control SY5Y-vec (black) cells. *p<0.05, ***p<0.0001 (C) NRG1 expression was analyzed in whole-cell lysates from SY5Y-NTRK1, SY5Y-NTRK2 and SY5Y-vec cells and in medium conditioned by the respective cell lines (CM). In whole-cell lysates β-actin served as the loading control. (D) *NTRK1* (green squares) and *NRG1* (blue dots) expression were re-analyzed using the R2 platform in previously generated exon array data from a representative cohort of 101 primary neuoblastomas [21]. p=4×10^−12^, r=0.622.

As a first step to examine interactions between *NTRK1*-expressing neuroblastoma cells with stromal cells of the peripheral nervous system, we reanalyzed gene expression data previously obtained from the neurotrophin recepter-expressing SY5Y cell culture model. The SY5Y neuroblastoma cell line was stably transfected with NTRK1, NTRK2 or a vector control to generate these models, designated SY5Y-NTRK1, SY5Y-NTRK2 and SY5Y-vec, respectively [18]. An automated search using the R2 platform provided a list of genes that were differentially expressed between the SY5Y-NTRK1, SY5Y-NTRK2 and SY5Y-vec cell models, and these were manually screened for genes previously reported to be involved in Schwann cell regulation. We identified *NRG1*, *FGF5*, *PDGFA* and *CXCL12* as four potential candidates that were also upregulated in SY5Y-NTRK1 cells (Fig. 1A). Notably, gene set enrichment analysis revealed an enrichment of genes belonging to the “glial cell differentiation” gene ontology (GO:0010001) in SY5Y-NTRK1 cells (compared with SY5Y-NTRK2 and SY5Y-vec cell models). This is the only glial cell-specific ontology subset, and includes both *NRG1* and *FGF5*. We confirmed the strong induction of all selected genes in SY5Y-NTRK1 cells using real-time RT-PCR (Fig. 1B). The NRG1 protein was previously shown to act as a chemokine for Schwann cell precursors, and strongly enhances migratory capacity and proliferation of adult rat Schwann cells *in vitro* [19, 20]. We examined NRG1 protein expression in cell lysates of and medium conditioned by our SY5Y cell model expressing either NTRK1 or NTRK2. NRG1 expression was restricted to cell lysates of NTRK1-positive neuroblastoma cells (Fig. 1C). Interestingly, NRG1 protein was also detected in medium conditioned by SY5Y-NTRK1 cells, but not SY5Y-NTRK2 or SY5Y-vec cells (Fig. 1C). Reanalyses of data from exon resolution mRNA arrays previously obtained from 101 primary neuroblastomas demonstrated a highly significant positive correlation between *NTRK1* and *NRG1* expression *in vivo* (Fig. 1D) [21]. Taken together, these data show that NTRK1 expression causes upregulation and secretion of the Schwann cell-stimulating factor, NRG1, *in vitro* and is strongly correlated with *NRG1* expression *in vivo*, suggesting a potential role for NTRK1 in the tumor-stroma dialog in neuroblastoma.

### NTRK1-positive neuroblastoma cells mediate proliferation and migration of Schwann cells by secreting NRG1

**Figure 2:**
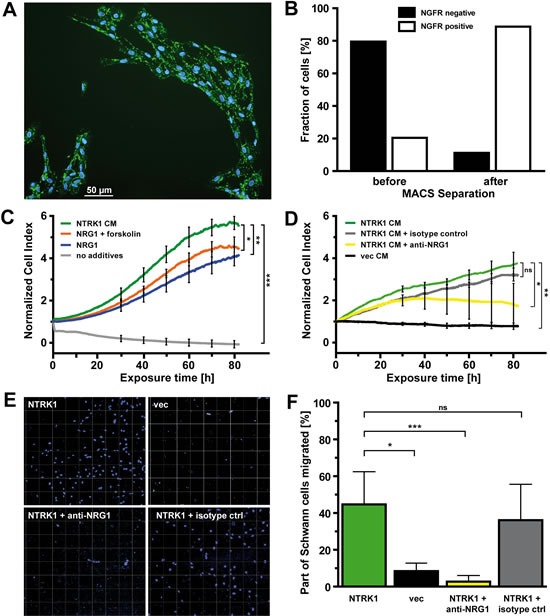
NTRK1-positive neuroblastoma cells attract Schwann cells and induce their proliferation by secreting NRG1 (A) Dual immunofluorescence staining using anti-rat NGFR primary antibody followed by goat anti-mouse IgG-FITC secondary antibody and DAPI counterstaining in Schwann cells in short-term culture from P3 rats (scale bar 50μm). (B) Bars represent the fractions of NGFR-positive and -negative cells from the total population of Schwann cells before and after positive selection using anti-rat NGFR primary antibody and rat anti-mouse IgG1 microbeads. (C) Growth curves of Schwann cells over a period of 80h as detected by electric cell-substrate impedance sensing in real-time following addition of medium conditioned by SY5Y-NTRK1 cells (NTRK1 CM), recombinant NRG1 + forskolin (NRG1 + forskolin), recombinant NRG1 (NRG1) or medium without additives (no additives). (D) Growth curves of Schwann cells over a period of 80h as detected by electric cell-substrate impedance sensing in real-time following addition of medium conditioned by SY5Y-NTRK1 cells (NTRK1 CM), medium conditioned by SY5Y-NTRK1 cells supplemented with anti-NRG1 isotype control (NTRK1 CM + isotype ctrl) or anti-NRG1 antibody (NTRK1 CM + anti-NRG1) or medium conditioned by SY5Y-vec cells (vec CM). For all growth curves in (C) and (D), cell index was normalized each at a base-time of 24h following plating of Schwann cells using an RTCA software-based algorithm. *p<0.05, **p<0.01, ***p<0.0001 (E) Schwann cells invading the membranes in a Boyden chamber assay 9 days after equal quantities of Schwann cells were plated in the upper chambers. Lower chambers contained SY5Y-NTRK1 cells cultured without additives (NTRK1), with anti-NRG1 antibody (NTRK1 + anti-NRG1) or with isotype control (NTRK1 + isotype ctrl) or SY5Y-vec cells (vec). Representative images are shown. Schwann cell nuclei are DAPI-stained. (F) Statistical analysis of Boyden chamber assays at conditions described in (E). Bars represent the percentage of Schwann cells that migrated through the membrane in relation to the number of cells initially seeded onto the upper membrane surface. ***p<0.0001, *p<0.05.

Schwann cells were isolated from sciatic nerves from P3 rats (Fig. 2A and), since the homogeneity and *ex vivo* viability of Schwann cells is highest at this developmental stage [22, 23]. Schwann cell proliferation rapidly decreased in the absence of supplemented control medium (Fig. 2C). Addition of recombinant NRG1 induced a proliferative response of Schwann cells, as has also been previously described [24, 25]. Growth rates were further increased by combined treatment of Schwann cells with NRG1 and forskolin, a strong Schwann cell mitogen that elevates intracellular cAMP levels (Fig. 2C) [26]. Interestingly, the highest growth rates with significantly enhanced proliferative activity of Schwann cells were achieved when medium conditioned by SY5Y-NTRK1 cells was added (Fig. 2C and fig. S1). This effect was NTRK1-specific, since proliferation of Schwann cells incubated with CM from SY5Y-vec control cells decreased to the level of medium without additions (Fig. 2C and, and fig. S1). To determine whether NRG1 is involved in mediating the proliferative effects of NTRK1 on Schwann cells, we neutralized NRG1 activity in the CM from SY5Y-NTRK1 cells by adding a polyclonal antibody against human NRG1. Inhibiting NRG1 in this way reduced Schwann cell proliferation by 50 % after three days compared to unmodified SY5Y-NTRK1 CM or SY5Y-NTRK1 CM supplemented with an isotype negative control antibody (Fig. 2D).

Since NRG1 has previously been demonstrated to also be essential for Schwann cell migration [27], we next examined the impact of NTRK1-expressing neuroblastoma cells on the migratory activity of Schwann cells. SY5Y-NTRK1 cells or SY5Y-vec cells were cultured in the lower chamber of a Boyden chamber, while Schwann cells were plated on the upper surface of the membrane. At day 9, 45 percent of Schwann cells migrated through the membrane when cultured with NTRK1-positive cells, while SY5Y-vec cells did not significantly induce Schwann cell migration (. 2E and F). Blocking NRG1 activity by adding the neutralizing anti-NRG1 antibody almost completely abolished migratory capacity of Schwann cells, while addition of the isotype control had no significant impact. These results demonstrate a crucial role of NTRK1 expression in neuroblastoma cells for stimulating and maintaining both the proliferative activity and migratory capacity of Schwann cells in the tumor stroma. NRG1, expressed and secreted by NTRK1-positive SY5Y neuroblastoma cells, was identified as a chemokine and a major mediator of mitogenic effects in Schwann cells.

### Schwann cells secrete NGF and promote differentiation of NTRK1-expressing neuroblastoma cells

**Figure 3:**
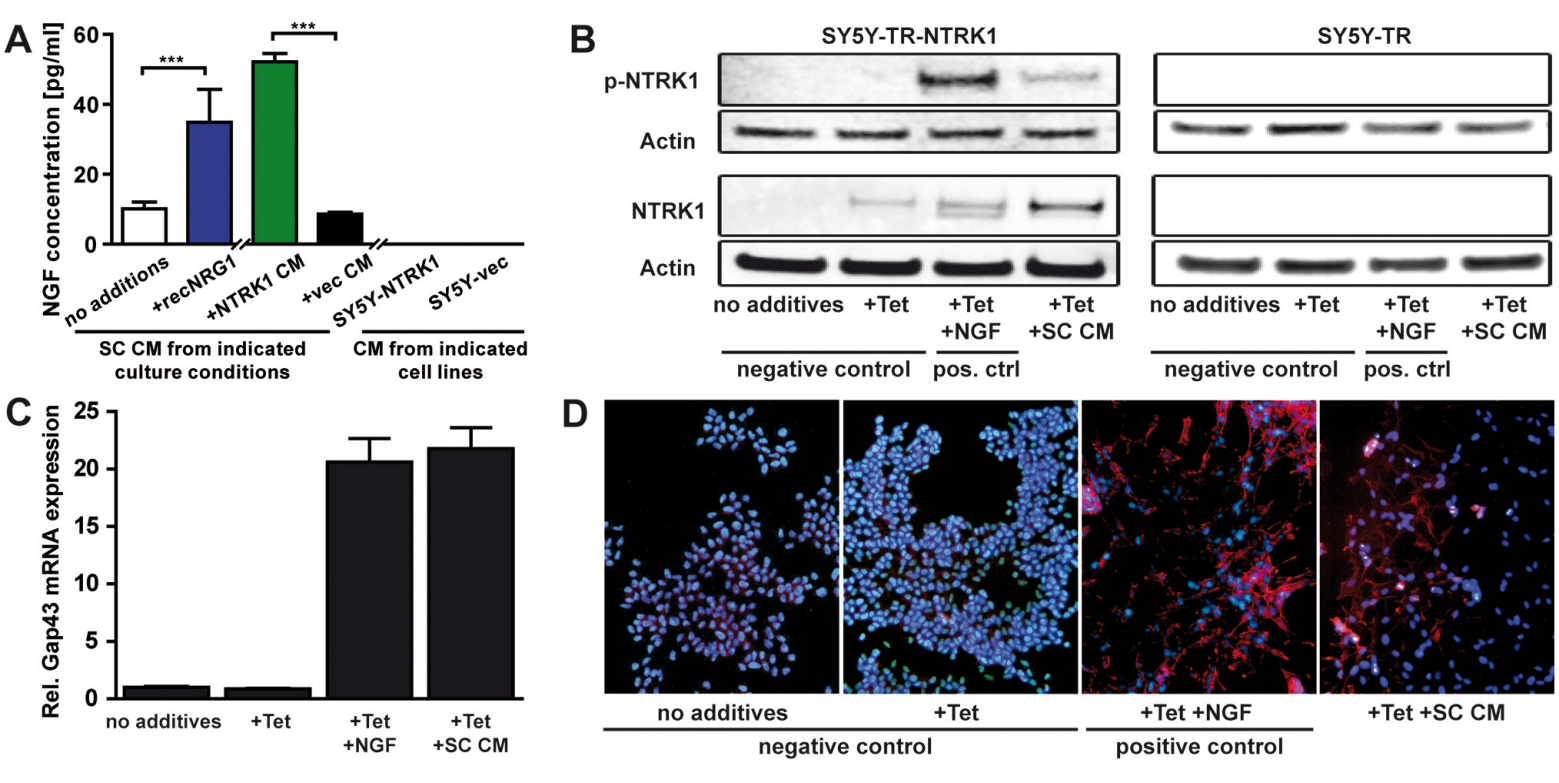
Schwann cells secrete NGF and promote differentiation of NTRK1-expressing neuroblastoma cells (A) Bars represent NGF concentrations (measured by ELISA) in *a priori* NGF-free medium conditioned by Schwann cells cultured without additives (no additives), with recombinant NRG1 (+recNRG1) or with medium conditioned by SY5Y-NTRK1 (+NTRK1 CM) or by SY5Y-vec (+vec CM) cells. NGF was not detected in medium conditioned by SY5Y-NTRK1 or SY5Y-vec cells. ***p<0.0001 (B) Detection of phosphorylated and total NTRK1 receptor expression (p-NTRK1 and NTRK1, respectively) by western blotting of whole-cell lysates of SY5Y cells with tetracycline-conditional expression of NTRK1 (SY5Y-TR-NTRK1) or control cells not expressing NTRK1 (SY5Y-TR). Prior to protein extraction, cells were exposed for 48h to tetracycline and Schwann cell-conditioned media (Tet + SC CM) or to nothing (no additives) or tetracycline alone (Tet) as negative controls, or to tetracycline and recombinant NGF (Tet + NGF) to induce and activate NTRK1 as a positive control. Experiments were repeated using control cells as negative controls. Actin expression was used as a loading control. (C-D) Differentiation of SY5Y-TR-NTRK1 cells after 5 days of tetracycline-induced NTRK1 expression in combination with stimulation by media conditioned by Schwann cells (Tet + SC CM) was assessed by measuring expression of the specific differentiation marker, *GAP43*, using real-time RT-PCR (C) and assessing phenotypic signs of differentiation in cultures stained with rhodamine-labeled phalloidin (red) and counterstained with DAPI (blue) to visualize the differentiation marker F-actin and nuclei, respectively (D). Cells were exposed to nothing (no additives) or tetracycline (Tet) alone as negative controls or to tetracycline and recombinant NGF in combination (Tet + NGF) to activate NTRK1 as a positive control.

Schwann cells were previously reported to express and secrete the NTRK1-specific ligand, NGF, which lead to peripheral nerve regeneration and axonal myelination as well as to neuroblastoma cell differentiation [28-30]. We assessed the conditions supporting NGF secretion into culture medium by Schwann cells (Fig. 3A). Basal NGF secretion by Schwann cells was markedly increased, when cells were cultured in medium supplemented with CM from SY5Y-NTRK1 cells compared to CM derived from SY5Y-vec cells. Interestingly, addition of recombinant NRG1 to Schwann cell cultures also induced NGF secretion by Schwann cells comparable to levels induced by adding SY5Y-NTRK1 CM. SY5Y-NTRK1 and SY5Y-vec cells did not secrete NGF. These findings strongly suggest that NRG1, being abundantly present in medium conditioned by SY5Y-NTRK1, is a crucial trigger of NGF production in Schwann cells.

Next we tested whether medium conditioned by Schwann cells was capable of activating the NGF-specific receptor, NTRK1, on SY5Y cells by evaluating receptor phosphorylation as a marker of activation. We used a vector system for conditional NTRK1 expression operating under a tetracycline repressor (SY5Y-TR-NTRK1) for these experiments since stable transfection of SY5Y cells with NTRK1 may result in auto-phosphorylation and subsequent auto-activity of the NTRK1 receptor [18]. Following induction of NTRK1 receptor expression by tetracycline, SY5Y-TR-NTRK1 and control cells were incubated with Schwann cell CM or recombinant NGF for 48h. Medium conditioned by Schwann cells could, indeed, cause effective NTRK1 receptor phosphorylation (Fig. 3B). Thus, NGF secreted by Schwann cells is functionally active. To further address the question whether Schwann cell CM may also recapitulate NGF-mediated differentiation of NTRK1-expressing neuroblastoma cells [8, 9], SY5Y-TR-NTRK1 cells were cultured in the presence of Schwann cell CM after NTRK1 induction. Medium conditioned by Schwann cells was as effective as recombinant NGF to increase expression of the differentiation marker, *GAP43*, in NTRK1-expressing SY5Y cells (Fig. 3C). Neuronal differentiation was also demonstrated by phalloidin-rhodamine staining of neurofilament formation in the NTRK1-expressing and Schwann cell CM-exposed SY5Y cells (Fig. 3D). Control experiments in SY5Y cells without NTRK1 expression (medium without additives) or with NTRK1 expression but no receptor activation (medium with tetracycline only) showed no onset of differentiation processes (Fig. 3C and) apart from a marginal level of microscopically detectable neurite outgrowth (data not shown). Taken together, we confirmed biosynthesis and subsequent secretion of NGF by cultured Schwann cells and the regulation of NGF secretion by NRG1. Our observations indicate that Schwann cell CM may activate the NTRK1 receptor to cause neuroblastoma cell differentiation. These findings suggest functional implications of Schwann cell-derived NGF on the development of maturing neuroblastoma subtypes relying on direct bidirectional interactions between tumor cells and their adjacent glia.

### NTRK1 expression in neuroblastoma cells adjacent to Schwann cells reduces tumor growth *in vivo*

**Figure 4:**
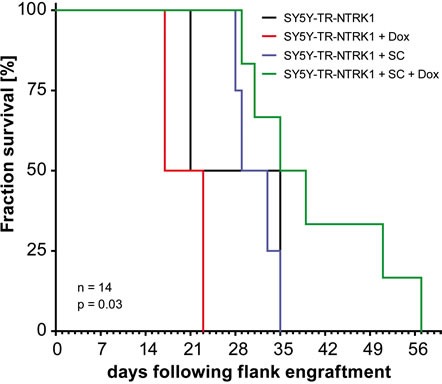
Schwann cells reduce proliferation of NTRK1-expresing neuroblastoma cells *in vivo* Kaplan-Meier analysis of xenograft tumor growth in nude mice injected with SY5Y-TR-NTRK1 cells mixed with Schwann cells and treated with doxycycline for NTRK1 induction (green curve) compared to control compositions: uninduced SY5Y-TR-NTRK1 cells without Schwann cells (black curve), induced SY5Y-TR-NTRK1 cells without Schwann cells (red curve), and uninduced SY5Y-TR-NTRK1 cells with Schwann cells (blue curve). (p=0.03, log-rank test).

We assessed the potential interaction of NTRK1-expressing neuroblastoma cells with Schwann cells in an *in vivo* experiment using a xenograft model of SY5Y cells in nude mice. As high constitutive expression of NTRK1 results in largely reduced tumorigenicity of this cell model, we again deployed the inducible model, SY5Y-TR-NTRK1, without NTRK1 expression in the absence of doxcycline. Nude mice (nu/nu) were subcutaneously injected with 1×10^6^ SY5Y-TR-NTRK1 cells mixed with 5×10^6^ Schwann cells in the flank to create mixed cell xenografts. NTRK1 expression was induced in SY5Y-TR-NTRK1 cells by adding doxycycline to the drinking water of mice. In order to discriminate between effects caused solely by NTRK1 induction or by the presence of Schwann cells, several control situations were used. Mice were injected with SY5Y-TR-NTRK1 cells alone and did not receive doxycycline in their drinking water so that no NTRK1 expression was induced, while other mice injected with SY5Y-TR-NTRK1 cells alone received doxycycline to induce NTRK1 expression. Mice were also injected with SY5Y-TR-NTRK1 and Schwann cell mixtures, but were not given doxycycline in their drinking water so that no NTRK1 expression was induced in the neuroblastic cells. Growth of xenografts containing neuroblastoma cells with induced NTRK1 receptor expression and mixed with Schwann cells was significantly delayed compared to tumors from control animals (p=0.03, log-rank test; Fig. 4).

Overall, our data demonstrate that progression of neuroblastoma growth *in vivo* may be delayed by simultaneously mimicking the most prominent genetic and histological hallmarks of neuroblastic tumors with favorable prognosis, NTRK1 expression and presence of Schwann cells.

## DISCUSSION

Increased Schwann cell content of neuroblastomas correlates with tumor maturation, and previous work has identified the Schwann cell secretome to be associated with antineuroblastic capability [30, 31]. Aiming to better understand the responsible underlying molecular mechanisms, we dissected the interplay between neuroblastoma and stroma cells and identified its principal regulators. Our study provides strong evidence that NTRK1-expressing neuroblastoma cells both attract and stimulate the proliferation of adjacent Schwann cells by secreting NRG1, which in turn leads to NGF-mediated differentiation of neuroblastic cells.

Schwann cells play a critical role in promoting neuronal regeneration and differentiation in the peripheral nervous system by expressing neurotrophic factors, including NGF [32, 33]. Kwiatkowski *et al.* observed neurite outgrowth in neuroblastoma cells when cultured in medium conditioned by Schwann cells, but failed to recapitulate these phenotypic changes *in vitro* by addition of recombinant NGF alone [30]. The authors continued to presume that the NGF/NTRK1 interaction contributed to differentiation *in vivo*, since defects in the NTRK1 receptor pathway of the employed cell lines hampered comparison with processes in less aggressive maturing neuroblastomas [34]. PEDF, after its identification in medium conditioned by Schwann cells, was suggested to be a multifunctional mediator of Schwann cell antitumor activity, since besides mediating anti-angiogenesis, it could also partly cause neurite outgrowth of neuroblastoma cells [35]. Our observations of only marginal neurite outgrowth in neuroblastoma cells without NTRK1 activation are consistent with these reports. In contrast, we demonstrated upregulation of genes involved in the differentiation process as well as appearance of neurofilaments, as strong indicators for maturation, to be strictly restricted to NTRK1-expressing cells. While factors secreted by Schwann cells appear to generally influence the neuroblastoma cell phenotype, we found the interaction of Schwann cell-derived NGF with neuroblastoma cells to be dependent on the expression of NTRK1 receptors on the neuroblastoma cell surface to induce a concerted maturation process of the latter *in vitro*.

**Figure 5:**
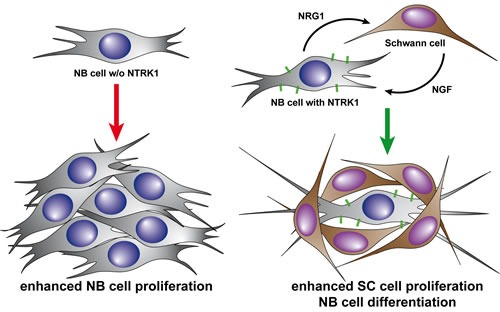
Diagramatic representation of our proposed model for bidirectional interaction between NTRK1-expressing neuroblastoma cells and adjacent Schwann cells Expression of NTRK1 on neuroblastic cells induces production of NRG1, which attracts Schwann cells. Infiltrating Schwann cells, in turn, secrete the specific ligand of NTRK1, NGF. Neuroblastic cells in this scenario (right) are triggered to differentiate by the activation of the NTRK1 receptor via NGF binding. In the absence of NTRK1 expression (left), neuroblastic cells do not differentiate and continue to proliferate, fueling the phenotype of progressive disease.

Based on our *in vitro* findings demonstrating that NTRK1-expressing neuroblastoma cells attract Schwann cells which, in turn, induce differentiation of the neuroblastic cells, we postulate that this constellation properly reflects the *in vivo* situation of biologically favorable neuroblastomas. These less aggressive tumors mostly express NTRK1 and are prone to spontaneous regression or differentiation, depending on the absence or presence of the specific ligand, NGF, in their microenvironment [36]. The proposed model of bidirectional interaction between NTRK1-expressing neuroblastoma cells and Schwann cells is depicted in Fig. 5 illustrating potential mechanisms which might explain why differentiated neuroblastomas with high NTRK1 expression have an elevated Schwann cell content, whereas poorly differentiated tumors with low NTRK1 expression are only marginally infiltrated by their stromal cells.

To test our hypothesis in an *in vivo* model, NTRK1-expressing tumor cells were co-injected with Schwann cells as mixed xenografts in nude mice, and tumor growth was compared to controls. Although we observed a significant reduction of xenograft tumor growth, the result did not fully recapitulate *in vitro* findings, most probably owing to the reduced proportion of Schwann cells in the mixed-cell xenografts by the end of the *in vivo* experiments (Fig. S2). It is likely that further mechanisms and key regulators of the neuroblastoma microenvironment remained unidentified and their holistic effects still need to be unraveled. Since interplay between Schwann cells and normal neurons is not restricted to NGF/NRG1 signals, but additionally depends on extracellular matrix interactions, cell-cell contact and various other signals [37], one might speculate that further costimulatory molecules (e.g. distinct growth factors or adhesion molecules) are needed to fully mimic the Schwann cell niche within an *in vivo* setting. In addition, the absolute number of Schwann cells or the ratio of Schwann cells to tumor cells set in the initial subcutaneous graft might be a crucial and limiting factor, since xenografted Schwann cells were not immortalized and definitely more susceptible to suboptimal growth conditions than neuroblastoma cell lines. Although immunocompromised mice were used in this experimental setting, the different species from which the cells originated (human neuroblastoma cells, rat Schwann cells) might be a further reason, why we could not fully recapitulate *in vivo* tumor regression induced by Schwann cells. Furthermore, expression of NTRK1, leading to reduced proliferation and angiogenesis [9, 38], constitutes a selective disadvantage for tumor cells. In consequence, NTRK1-expressing subclones have previously been demonstrated to downregulate NTRK1 expression or to be overgrown by neuroblastoma cells not expressing NRTK1 in the *in vivo* setting [38]. Indeed, NTRK1 expression was no longer detected in mixed neuroblastoma-Schwann cell xenografts at the end of our *in vivo* experiment (data not shown). According to our proposed model of bidirectional interaction, this might explain the diminished content of vital glia within the mixed-cell xenografts, since the Schwann cell effector molecule, NRG1, is not secreted by NTRK1-negative neuroblastoma cells.

Schwann cells are the most widely distributed type of neural crest-derived cells in the body, and are capable of invading different compartments and subsequently becoming essential components of specific neural and nonneural cell niches [39]. In return, affected cells exert different modes of action on their supporting Schwannian stroma, varying from induction or repression of proliferation to stimulation of differentiation or modification of migration [40]. NRG1 secreted by peripheral axons has previously been identified as an essential factor for Schwann cell proliferation and migration [27, 41, 42]. Here we demonstrate that NRG1 is also a predominant effector molecule of NTRK1-expressing neuroblastoma cells regulating communication with Schwann cells. Strikingly, expression of the NRG1 receptors, the ERBB2 and ERBB3 tyrosine kinases, have very recently been shown to be a marker of neuroblastic tumors with favorable prognosis [43].

It has been controversially discussed whether intratumoral Schwann cells should be considered as tumor cells differentiating along the glial cell lineage or represent an independent cellular population invading the tumor from the surrounding microenvironment [44-47]. Our study clearly defines intercellular communication mechanisms between tumor cells and Schwann cells, constituting the glia of the neuroblastoma microenvironment. This cross-talk leads to migration of Schwann cells and differentiation of neuroblastoma cells, thus, resembling similar phenomena observed in maturing neuroblastomas. These results are in line with the model for intratumoral Schwann cell invasion from outside the tumor rather than derivation from a common neoplastic stem cell within the tumor [16, 47].

We have recently shown that cross-talk between neuroblastoma and immune effector cells is significantly influenced by expression of NTRK1 [17]. The present study extends our knowledge of NTRK1-regulated processes to the field of tumor-stroma interactions. Mutual interaction of NTRK1-expressing neuroblastomas with Schwann cells via the NRG1/NGF-axis helps to explain both the high Schwann cell infiltration in NTRK1-positive primary neuroblastomas and the absence of Schwann cells in NTRK1-negative neuroblastomas. Paracrine communication within this complex tumor are certainly not limited to the NRG1 and NGF effector molecules with the NTRK1 receptor, since the immune system and angiogenic tumor supply also contribute to overall neuroblastoma clinical presentation and aggressiveness. Nevertheless, interrelation between the NTRK1-positive neuroblastic cell subpopulation and adjacent Schwann cells proposed here are likely to mirror conditions found in maturing neuroblastic tumors. Thus, our results point to two potentially important clinical implications: i) therapeutic induction of NTRK1 might be a promising approach to create a less malignant tumor phenotype that requires less intensive therapy, and ii) in addition to developing new therapies targeting the tumor, strategies should also focus on enhancing components in the microenvironment that inhibit tumor growth.

## MATERIALS AND METHODS

### mRNA expression analyses

Microarray expression profiles of the human SY5Y cell line expressing either NTRK1/TrkA or NTRK2/TrkB were generated previously [18], and analyzed for expression of genes involved in Schwann cell regulation. *In vivo* expression data were previously obtained from 101 primary neuroblastomas using exon arrays [21]. Microarray data have been submitted to the GEO database (acc. nos. GSE18409 and GSE32664). The R2 platform (http://hgserver1.amc.nl/cgi-bin/r2/main.cgi) was used for microarray analyses and visualization. For validation by real-time RT-PCR, total RNA was isolated from cells using the RNeasyMini kit (Qiagen, Hilden, Germany) and cDNA synthesis was performed using the SuperScript reverse transcription kit (Invitrogen, Darmstadt, Germany). *NRG1*, *FGF5*, *PDGFA*, *CXCL12* and *GAP43* expression was monitored by real-time PCR using “Assays on Demand” (Applied Biosystems, Carlsbad, CA, USA). Expression values were normalized to the geometric mean of the three housekeeping genes, *GAPDH*, *UBC* and *HPRT* [48].

### Protein analyses and antibodies

Either whole-cell protein extracts or concentrated conditioned medium was separated on gradient (4-12%) SDS-PAGE and used for western blotting. Immunoblot analysis was performed using anti-NRG1 (Cat.# AF2015, R&D, Minneapolis, MN, USA), 1:500; anti-phospho-NTRK1 (Cat.# 4621, Cell Signaling, Danvers, MA, USA), 1:1 000; anti-β-actin (Sigma-Aldrich, St Louis, MO, USA), 1:5 000, and HRP-conjugated goat anti-mouse or anti-rabbit secondary antibodies (GE Healthcare). ImageJ 1.42q (W. Rasband, NIH, Bethesda, MD, USA) was used to quantify signal intensities.

### Neuroblastoma cell culture and conditioned medium

The SH-SY5Y neuroblastoma cell line (further designated as SY5Y) and their derivative clones were cultivated as previously described [18]. Cell line identity was authenticated by the DSMZ (Braunschweig, Germany). Stable expression of NTRK1/TrkA or NTRK2/TrkB expression in SY5Y has been described previously.[18] Inducible NTRK1 expression in SY5Y was achieved by cloning human NTRK1/TrkA cDNA into the pT-REx-DEST30 vector (Invitrogen, Carlsbad, CA, USA) for tetracycline-conditional expression [17]. In brief, the SY5Y cell line was sequentially transfected with pT-REx-DEST30 then pcDNA6/TR (Invitrogen), harboring the tetracycline repressor gene. Single cell clones were selected by limiting dilution in medium containing blasticidine and G418 (Invitrogen). NTRK1/TrkA induction was realized by adding 1μg tetracycline per ml medium. For generation of conditioned medium (CM), cell cultures were washed with PBS and incubated 48h with serum-free medium. CM was centrifuged after collection, and complete protease inhibitor cocktail (Roche, Mannheim, Germany) was added before concentration using Amicon Ultra-15 centrifugal filter units (Millipore, Billerica, MA, USA). Receptor activation was achieved with 100ng/ml recombinant NGF (R&D Systems, Minneapolis, MN, USA) in both the inducible and constitutive systems as a positive control. SY5Y cells transfected with pcDNA6/TR only served as a control in experiments using the tetracycline-inducible cell culture model.

### Schwann cell isolation, culturing, conditioned medium and detection of NGF

Schwann cells were isolated from explanted sciatic nerves from P3 Sprague-Dawley rats by a series of enzymatic trituration and cultivation steps, including the positive selection of Schwann cells based on NGFR expression (modified from [49, 50]). In brief, the epineurium was carefully released using an operating microscope. Nerve fascicles were washed gently with HBSS (Invitrogen) and incubated with PBS (Invitrogen) supplemented with liberase DL (Roche) at 37°C and 5% CO_2_ for a maximum of 30 minutes. Enzymatic digestion was stopped by adding culture medium (see below). Schwann cells were positively selected using the MACS MicroBeads® technique (Miltenyi, Bergisch Gladbach, Germany) with anti-rat NGFR primary antibody (Millipore) and rat anti-mouse IgG1 MicroBeads (Miltenyi). Approximately 1-5×10^6^ Schwann cells were plated per well onto poly-L-lysine- coated (Biochrom, Berlin, Germany) 6-well plates in DMEM supplemented with 10% FCS, 200mM L-glutamine, 1% penicillin/streptomycin (Invitrogen) and 100mM sodium pyruvate (Sigma-Aldrich). For Schwann cell expansion, medium was supplemented with 1μM forskolin (Merck, Darmstadt, Germany) and/or 12.5μg/ml of recombinant NRG1 (R&D Systems). Conditioned medium was prepared as described for neuroblastoma cell lines and clones, but culture medium supplemented with the NGF-free serum substitute, Panexin NTA (PAN-Biotech, Aidenbach, Germany), instead of FCS was used. NGF was quantified using Emax® ImmunoAssay Systems (Promega, Mannheim, Germany). Schwann cell conditioned medium was concentrated as described above and added to respective neuroblastoma media at a 1:10 ratio in all experiments.

### Immunostaining and phalloidin-based staining

Immunostaining of Schwann cells was performed using anti-rat NGFR primary antibody (Cat.# AB1554, Millipore) followed by goat anti-mouse IgG-FITC secondary antibody (Southern Biotech, Birmingham, AL, USA). Phalloidin conjugated to tetramethylrhodamine (TRITC, Sigma-Aldrich) was used for detection of the cytoskeletal differentiation marker F-actin in SH-SY5Y-TR-NTRK1 cells. Nuclei were counterstained with 4′,6-diamidino-2-phenylindole (DAPI, Invitrogen).

### Cell proliferation analyses

Schwann cell proliferation was analyzed in real-time using the xCELLigence^TM^ system (ACEA Biosciences, CA, USA). Briefly, 3 500 cells were seeded into 96-well plates, and changes in cellular indices (CI) as a surrogate marker for cell proliferation were detected after addition of conditioned media, 12.5μg/ml recombinant NRG1 with or without forskolin (Merck), a polyclonal antibody specific for human NRG1 (4μg/ml, cat.# AF2015, R&D Systems) or an isotype control for the anti-NRG1 antibody (8μg/ml, cat.# 02-6202, Invitrogen). CI values obtained from electric cell-substrate impedance sensing were calculated and normalized to facilitate comparison using the RTCA real-time cell analyzer software (ACEA Biosciences).

### Cell migration analysis

Migration was evaluated in a Boyden chamber 6-well format (BD Bioscience) that utilized poly-L-lysine-coated polycarbonate membranes. SY5Y-NTRK1 or SY5Yvec cells (10^4^ each) were placed in the lower compartment containing cell culture medium supplemented with 10% FCS. In the upper compartment, 2.5×10^4^ or 5×10^4^ Schwann cells were added in Schwann cell culture medium. The membrane was removed after 9 days, and Schwann cells were dislodged from the upper filter surface using a rubber policeman. Cells that had migrated were stained with DAPI (Invitrogen) and counted using fluorescence microscopy. Experiments were carried out in triplicate and were repeated three times.

### Growth of xenograft tumors in nude mice

Mouse experiments were carried out in accordance with the principles of laboratory animal care (NIH publication NO. 86–23, revised 1985) and the German Animal Welfare Act and were approved by the local animal ethics committees (Az. 8.87-50.10.37.09.202). SY5Y-TR-NTRK1 neuroblastoma cells were cultivated until they reached 80% confluency. Schwann cells were isolated from the sciatic nerve of P3 rats as described above and expanded in culture medium supplemented with recombinant NRG1 for several days. A mixture of 1×10^6^ SY5Y-TR-NTRK1 cells and 5×10^6^ Schwann cells was suspended in 100 μl matrigel (BD Bioscience), and subcutaneously inoculated in the flank of six-week old female athymic NCR (nu/nu) mice. Doxycycline, a semi-synthetic tetracycline which is better tolerated by the animals, was added to the drinking water (1mg/ml, in flasks impervious to light). This approach assured an average intake of 5mg doxycycline per mouse per day, thus, reaching *in vivo* drug levels proven to be capable of NTRK1 induction. Treatment was started three days prior to xenograft injection (experimental group = 8 mice). Control animals received only SY5Y-TR-NTRK1 cells with (2 mice) or without NTRK1 induction (2 mice) or SY5Y-TR-NTRK1 cells mixed with Schwann cells, but without NTRK1 induction (4 mice). Mice were sacrificed when tumor volume reached 1cm^3^ according to institutional guidelines.

### Statistics

Microarray data obtained from the SH-SY5Y cell culture model and from primary neuroblastomas were reanalyzed using the R2 platform (http://hgserver1.amc.nl/cgi-bin/r2/main.cgi). Real-time RT-PCR data were analyzed using qBase 1.4 (Biogazelle, Ghent, Belgium). SPSS 18.0 (IBM, Ehningen, Germany) was used to conduct Student's t-tests (one-sided for Schwann cell proliferation and two-sided for all other analyses) for all interval variables. All error bars represent mean +/− SD, if not otherwise indicated. Graph Pad Prism 5.0 (GraphPad Software, La Jolla, CA, USA) was used for visualization and Kaplan-Meier survival analysis with log-rank statistics.

## Supplementary Material FIGURES


