# Supplementary material for: Neuroblastoma in dialog with its stroma: NTRK1 is a regulator of cellular cross-talk with Schwann cells
Source: Oncotarget. 2014 Oct 21;5(22):11180–92. doi: 10.18632/oncotarget.2611 (PMC4294349; doi:10.18632/oncotarget.2611)
Supplement: Supplementary file 1 [file oncotarget-05-11180-s001.pdf]

Neuroblastoma in dialog with its stroma: NTRK1 is a regulator of cellular cross-talk with Schwann cells

Supplementary Material

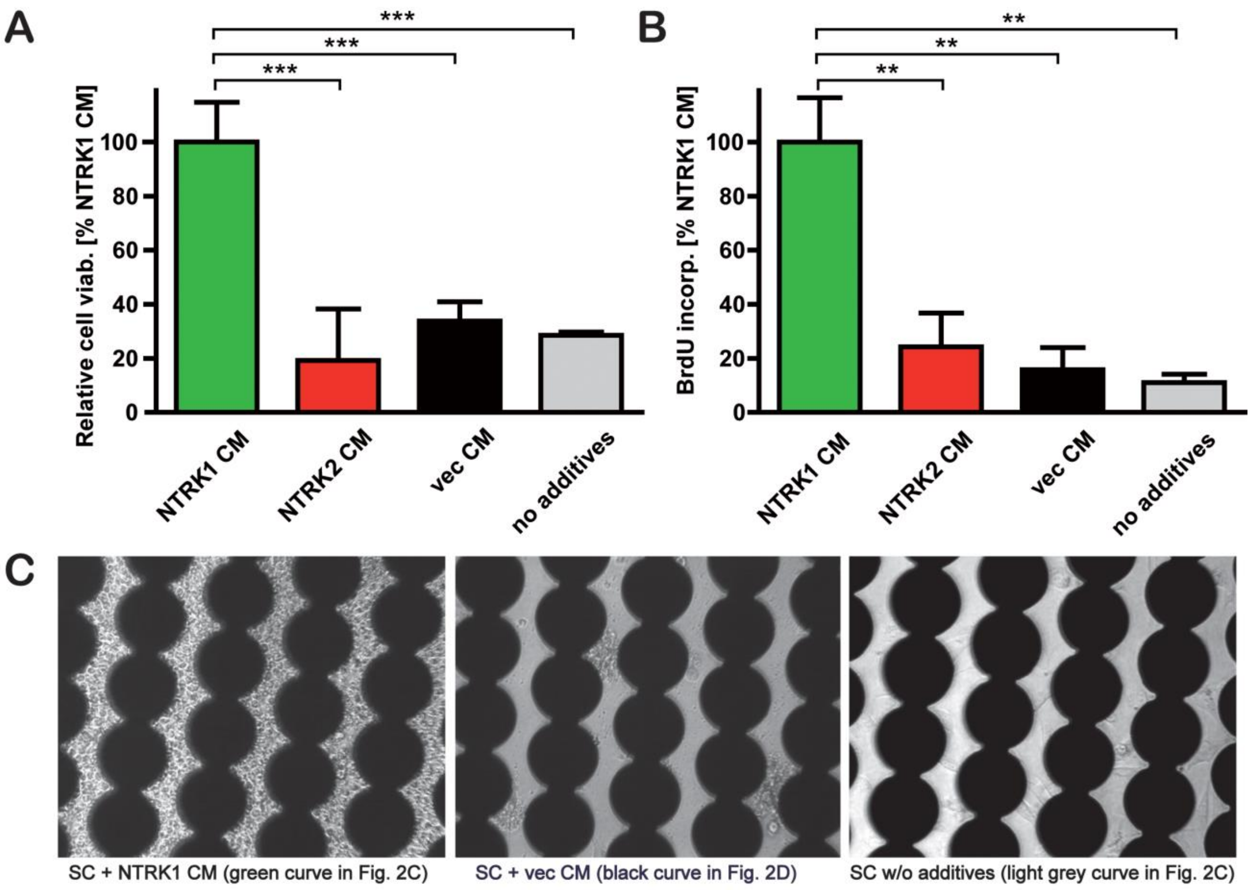

Figure S1: NTRK1-expressing neuroblastoma cells sustain Schwann cell viability and induce their proliferation. **(A)** Cell viability was assessed using the MTT assay after incubating Schwann cells with the indicated media for 72h. Extinction relative to Schwann cells incubated with media conditioned by SY5Y-NTRK1 (SY5Y-NTRK1 CM) is shown. ***p<0.0001 **(B)** Schwann cell proliferation was measured by BrdU ELISA after culturing 72h in the indicated media. Extinction relative to Schwann cells incubated with conditioned media (SY5Y-NTRK1 CM) is shown. **p<0.01 **(C)** Micrographs of representative areas of the xCELLigence culture plates from experiments that generated data displayed in Fig. 2C and D showing cell density at

termination after 80h of culture time. Schwann cells incubated with medium conditioned by SY5Y-NTRK1 cells (SY5Y-NTRK1 CM) have greatly expanded in number, while few Schwann cells remain after incubation with medium conditioned by SY5Y-vec cells (SY5Y-vec CM) or control medium.

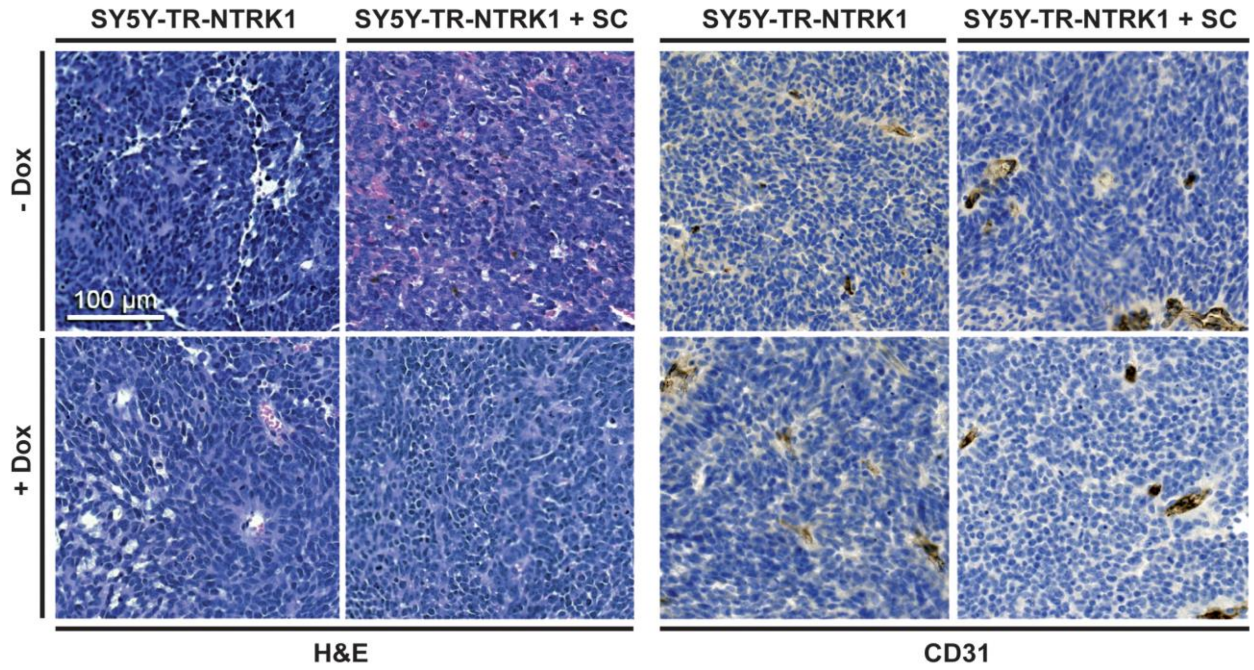

Figure S2: Selected micrographs show representative immunostaining for CD31 in addition to hematoxylin & eosin staining (HE) for xenografts of the experimental group (SY5Y-TR-NTRK1 + Dox + SC) and all control conditions by the end of the *in vivo* experiment.
